# Combined effects of chronic high glucose and fluoride exposure on kidney cells: exploratory in vitro and in vivo study

**DOI:** 10.1007/s10735-025-10670-6

**Published:** 2026-01-12

**Authors:** Laura Ribeiro, Heloísa Aparecida Barbosa da Silva Pereira, Juliana Sanches Trevizol, Aislan Quintiliano Delgado, Tânia Mary Cestari, Marília Afonso Rabelo Buzalaf, Rodrigo Cardoso de Oliveira, Claudia Cristina Biguetti

**Affiliations:** 1 Department of Biological Sciences, University of São Paulo – Bauru School of Dentistry, Alameda Octávio Pinheiro Brisolla, 9-75, Bauru, São Paulo, 1712-901 Brazil; 2 School of Podiatric Medicine, The University of Texas Rio Grande Valley, Harlingen, TX USA; 3 Institute of Biosciences, São Paulo State University, Botucatu, São Paulo, Brazil

**Keywords:** Fluoride, Diabetes, Kidney, Hyperglycemia

## Abstract

Fluoride is commonly used in dentistry to prevent dental caries, however, excessive exposure may pose risks to soft tissues, particularly the kidneys, which are responsible for approximately 60% of fluoride excretion. Diabetic nephropathy, a major complication of diabetes mellitus, may share pathogenic pathways with fluoride-induced renal toxicity. However, the combined effects of chronic hyperglycemia and fluoride exposure on kidney cells remain poorly understood. This study investigated the effects of fluoride on murine renal tubular epithelial cells (M-1) and on murine kidneys under normal and hyperglycemic conditions. M-1 cells were cultured under high-glucose conditions (22.5 mM) and/or treated with fluoride (1 µM or 5 µM) for 24 to 72 h. Diabetic C57BL/6J mice received drinking water containing fluoride (10 mgF/L or 50 mgF/L) for 21 days. Evaluations included cell viability and morphology in vitro, collagen deposition in renal tissue by birefringence analysis, and expression of the kidney injury marker KIM-1 by immunofluorescence in both models. M-1 cells exposed to fluoride alone showed increased viability at 72 h, while KIM-1 expression was elevated in high-glucose and high-glucose + 1 µM fluoride conditions, suggesting a stress or adaptive response. In diabetic mice, glomerular collagen accumulation, indicative of early fibrosis, was observed but attenuated by fluoride treatment. However, high KIM-1 levels in fluoride-treated diabetic mice, particularly at the higher dose, indicated potential kidney injury. These results highlight a complex, dual role of F in the diabetic kidney, potentially protecting against glomerular fibrosis while exacerbating tubular injury at high doses. Careful monitoring of F exposure is needed, especially in areas with endemic fluorosis and high chronic kidney disease risk.

**Supplementary Information:**

The online version contains supplementary material available at 10.1007/s10735-025-10670-6.

## Introduction

Fluoride (F) is a naturally occurring element and is commonly found in a variety of sources, including dental products, beverages, foods, and most notably through fluoridated water. Indeed, water fluoridation, administered by public water systems, stands as one of the most significant public health achievements of the twentieth century, and is widely recognized for its effectiveness in preventing dental caries (Dionizio et al. 2020). The World Health Organization (WHO) recommends a guideline of 1.5 mg/L for F in drinking water, while the American Dental Association (ADA) and the Department of Health and Human services suggest a lower guideline level of 0.7 mg/L (Arakawa et al. 2009; Meenakshi et al. 2024), maintaining optimal fluoride concentrations is critical to maximizing dental benefits while minimizing potential adverse effects.

While lower chronic levels of F can be beneficial in preventing caries, exposure exceeding the recommended WHO limits raises concerns about its potential adverse effects on calcified and soft tissues (Barbier et al., 2010; Wei et al. 2014). Once absorbed in the gastrointestinal tract, F is distributed throughout the body, reaching all tissues (Lupo et al. 2011). Due to its strong affinity for calcified tissues, F predominantly accumulates in bone, dentin, and enamel, with any excess being excreted primarily through urine (Saad et al. 2022). The effects of chronic exposure of F on hard tissues are well-documented, with evidence showing that acute or chronic consumption of high concentrations can cause dental and skeletal fluorosis, characterized by hypomineralization of dental structures and brittle bones, respectively (Arakawa et al. 2009; Meenakshi et al. 2024). However, a small portion of F is absorbed by soft tissues, whereas a high F exposure can lead to non-skeletal fluorosis. Indeed, it has been shown that F can affect a wide range of soft tissues such as the kidneys, but the influence of F on these organ cells metabolism remains incompletely understood (Meenakshi et al. 2024).

Previous studies have shown that non-skeletal fluorosis may disrupt crucial metabolic pathways and inhibit various enzymes, including those involved in the glycolytic pathway (Barbier et al., 2010). For example, F can affect glucose metabolism, with prolonged exposure potentially leading to insulin resistance and impaired glucose tolerance, it can interfere with the function of pancreatic beta cells and may also influence the insulin signaling pathway, contributing to insulin resistance (Meenakshi et al. 2024). In addition, some studies performed in murine models have found that F intake has been shown to increase insulin sensitivity in tissues such as the kidneys, liver, lung and spleen (Lupo et al. 2011; Trivedi et al. 1993). Other studies also performed in murine models have demonstrated that both chronic and acute ingestion of F alters protein expression in the liver, muscle, kidney and intestines (Dionizio et al. 2019; Kobayashi et al. 2009; Lima Leite et al. 2014; Lobo et al. 2015; Pereira et al. 2018). Amongst these organs, kidneys are of particular importance as they are responsible for 60% of the total daily F absorbed elimination, making them approximately 4–5 times more susceptible to F toxicity than other organs (Kobayashi et al. 2009).

These findings highlight the clinical significance of excessive fluoride intake, particularly for population with metabolic disorders such as Diabetes Mellitus (DM), specifically type 2 diabetes mellitus (T2DM). DM is a persistent metabolic disorder marked by chronic hyperglycemia, resulting either from insufficient insulin production (type 1) or the unresponsiveness of insulin receptors (type 2) (Hossain et al. 2024; Meenakshi et al. 2024). Type 1 diabetes mellitus (T1DM) primarily arises from autoimmune destruction of the pancreatic beta cells, while T2DM is significantly influenced by genetic factors, diet, obesity, and sedentary lifestyles (Hossain et al. 2024). The international Diabetes Federation (IDF) reports that more than 500 million people worldwide are affected by diabetes, with projections indicating a 30% increase in prevalence by 2045 (IDF Diabetes Atlas Group, 2011). Amongst several complications, DM remains the leading cause of chronic kidney disease (CKD) globally (Merchant and Klein 2007). Diabetic nephropathy (DN), which is the primary contributor to CKD in diabetic patients, typically results from persistent hyperglycemia, metabolic disturbances and genetic factors (Merchant and Klein 2007). The global incidence of DN has been on the rise in recent years, largely driven by the increasing prevalence of T2DM, with 35–40% of individuals with DM expected to develop DN (Merchant and Klein 2007). In this regards, individuals with DM residing in endemic fluorosis areas may be at higher risk of exacerbating existing diabetic nephropathy or compromise disease management through soft tissue toxicity mechanisms (Meenakshi et al. 2024).

Kidney injury molecule-1 (KIM-1) is a type 1 transmembrane glycoprotein that is upregulated in response to ischemic or toxic insults to the kidneys. Its expression is minimal or absent in health kidney, however, following injury, KIM-1 is upregulated especially by the kidney proximal tubular epithelial cells (Humphreys et al. 2013; Ichimura et al. 2004). Due to its high sensitivity and specificity as a marker of tubular damage, KIM-1 has been recognized as a reliable indicator of renal injury. In this project, KIM-1 was chosen as a key biomarker because its upregulation not only allows early detection of kidney injury but also reflects ongoing inflammatory and fibrotic processes (Humphreys et al. 2013; Huo et al. 2010).

Given increased susceptibility of patients with DM in developing CKD, it is essential to investigate the role of F toxicity in kidney tissues. Interestingly, the concentrations ratios of F in kidney can vary significantly depending on the context of exposure, making this organ particularly susceptible to F-induced damage depending the dose concentration of F (Barbier et al., 2010). Indeed, an epidemiological study conducted on a Mexican population indicated that certain kidney injury biomarkers, such as KIM-1, is potentially associated with chronic environmental F exposure and tubular cell damage in vulnerable groups (Jiménez-Córdova et al. 2018). Despite numerous reports in the literature regarding F toxicity in various tissues, our understanding of the cellular and molecular mechanism responsible for these effects in kidneys remains limited, particularly regarding dose concentrations and their combined effects in a chronic hyperglycemic environment. Thus, the aim of the present study is to evaluate fluorides influence on kidney injury marker (KIM-1), cell viability and histological parameters under chronic hyperglycemic conditions using in vitro and in vivo models.

## Materials and methods

### Cell culture and treatments

M-1 CRL-2038 cell line, derived from cortical collecting renal duct epithelial cells was acquired from ATCC (American Type Culture Collection). This cell line was established from normal renal tissues of *Mus musculus* transgenic mice. Cells were cultured in 25 cm^2^ plastic bottles containing DMEM F12 (D8437, Sigma-Aldrich) supplemented with 10% fetal bovine serum (FB12999102, Fisher Scientific). The cells were maintained in an incubator at 37 °C with 5% CO_2_/air atmosphere, and the culture medium was changed every two days. M-1 cells were seeded in regular glucose DMEM F12 medium (17.5 mM glucose), as control (Ctrl) or high-glucose levels (22.5 mM glucose) to mimic hyperglycemic environment (HG group) the choice of 22.5 mM glucose to simulate hyperglycemia was based on previous studies in which this concentration induced changes consistent with hyperglycemic state (Gstraunthaler et al. 1999; Mei et al. 2022). An osmolarity control group was cultured with regular DMEM F12 added to 5 mM mannitol, to reach 22.5 mM osmolarity (Mei et al. 2022). To evaluate the effects of F, M-1 cells were treated with sodium fluoride (NaF) at concentrations of 1 µM (F1) or 5 µM (F5). These concentrations were selected based on F levels reported in human serum and urine, reflecting exposure in regions with normal to low F levels (1–2.4 mgF/L) as well as areas with elevated F exposure 910–80 mgF/L) (Mendoza-Schulz et al. 2009; Ribeiro et al. 2017). The treatment preparation followed the protocol previously described by Buzalaf et al. (2001). Initially, a stock solution of 40 mM NaF (F1019, LabSynth) was prepared by diluting it in 1 × PBS. This solution was then filtered inside a laminar flow hood and stored at −20 °C. On the day of the experiment, the stock solution was diluted to the desired concentrations (1 µM or 5 µM) using DMEM F12 supplemented with 10% FBS and high-glucose DMEM F12 supplement with 10% FBS. Cells were seeded with regular DMEM F12 medium, allowed to attach overnight and then they were treated with either HG, F, or a combination of both for the periods of 24 h, 48 h, and/or 72 h. All treatment groups are demonstrated in Table 1. For the in vitro experiments, at least three independent experiments were performed, each with four technical replicates per condition, ensuring reproducibility of the results.

**Table 1:** In vitro group distribution according to each experimental condition

Group	M-1 cell	Exposure scenario
NC	Internal control	–
MNT 5 mM	Osmolarity control + 5 mM Mannitol	–
MNT 7.5 mM	Osmolarity control + 7.5 mM Mannitol	–
Ctrl	DMEM F12 and 0 NaF	–
F1	DMEM F12 + NaF 1 µM	Normoglycemic levels + 1 mgF/L (aligned with WHO fluoridation guidelines)
F5	DMEM F12 + NaF 5 µM	Normoglycemic levels + 5 mgF/L (representing endemic F areas with naturally elevated drinking water levels)
HG	22.5 mM glucose	Chronic hyperglycemia (glucose > 200 mg/dL) + 0 mgF/L
F1 + HG	22.5 mM glucose and NaF 1 µM	Chronic hyperglycemia (glucose > 200 mg/dL) + 1 mgF/L (aligned with WHO fluoridation guidelines)
F5 + HG	22.5 mM glucose and NaF 5 µM	Chronic hyperglycemia (glucose > 200 mg/dL) + 5 mgF/L (representing endemic F areas with naturally elevated drinking water levels)

### Animals and experimental design

Animal experiments were performed upon approval by the local Institutional Committee for Animal Care and Use (CEEPA-FOB/USP 001/2019 and CEEPA-FOB/USP 003/2022) and according to recommendations in the Guide for the Care and use of Laboratory Animals of the Institute of Health (Institute of Laboratory Animal Resources (U.S), 2021). Forty-two-day-old C57BL/6 and C57BL/6 J male mice were obtained from the University of São Paulo, after a 7-day acclimatization period under controlled conditions. The mice were housed under standard conditions of temperature (22–14 °C), humidity (60%), and a 12-h light/dark cycle, with food and water provided *ab libitum* both before and during the experimental period, the animals were housed in groups of 3/cage. Body weight of the animals were monitored weekly from week 0 till week 12 end of experimental period. The C57BL/6 J strain was selected for its genetic well-documented predisposition to insulin resistance and impaired glucose metabolism, making it suitable for modeling T2DM, while the C57BL/6 strain represents a normoglycemic baseline without such genetic susceptibility (Srinivasan and Ramarao, 2004). Therefore, the comparison between these strains was designed to reflect the biological contrast between metabolically health and diabetes-prone phenotypes, rather than to isolate F effects within a single genetic background. 30 C57BL/6 J mice were fed a high-fat diet (< 1 mg/kg F) for at least 8 weeks until they reached 30 g (Gilbert et al. 2011). The number of animals per group (n = 10) was determined based on previous studies using similar protocols for diabetes and chronic fluoride exposure, and was also the sample size appropriate by the local Institutional Committee for Animal Care and Use (CEEPA) (Dionizio et al. 2019; Gilbert et al. 2011; Pereira et al. 2013). Subsequently, the complete phenotype of DM was achieved via intraperitoneal injections of streptozotocin (STZ) at 40 mg/kg for 3 consecutive days. DM was confirmed by fasting glycemia levels exceeding 200 mg/dL within a week post the last injection of STZ. Blood glucose levels were monitored one week before and one week after STZ induction using a glucometer (Accu-Chek Performa, Roche Diagnostics, Mannheim, Germany). Blood samples were collected from the caudal vein of the animals. The diabetic mice were then provided with NaF-supplemented water for 21 days and divided into three groups (n = 10) based on F concentrations. The concentrations chosen for this study were based on regions with low to normal F level (1–2.4 mgF/L) and areas with elevated F levels (10–80 mgF/L) (Mendoza-Schulz et al. 2009). Given that F metabolism in rodents is 5–10 times faster than in humans the concentrations used in the animal model of 10 and 50mgF/L correspond to 1 mgF/L and 5 mgF/L in humans, respectively (Buzalaf et al. 2001). The F solutions were prepared according to the desired concentration. For the 10 mgF/L solution, 110.52 mg of NaF (F1019, LabSynth) were weighted and diluted in 5 L of Milli-Q water. For the 50 mgF/L solution, 552.6 mg of NaF were used under the same conditions. The prepared solution were stored in plastic bottles at 4 °C and provided to the animals as drinking water. New solution were prepared every two weeks or whenever needed. The F concentration in the artificially F water was verified as previously described by Pereira et al. (2013). Measurements of F ions were carried out using an ion-selective electrode (Accumet, #13–620-79) connected to a potentiometer (Orion Research, Model EA940). F standards ranging from 100, 50, 10, and 1 ppm F were prepared with the Orion™ ISE Calibration Standards (Orion 940,907, Thermo Scientific) in triplicate standards. Finally, electrode potential (mV) were converted to F concentrations of (µg F) using a standard calibration curve with a correlation of coefficient of r > 0.99. A summary of the animal groups is provided in Table 2. To establish a proper non-diabetic control group, ten male C57BL/6 mice were included in the study. These animals were maintained under the same conditions and experimental timeline as the treated C57BL/6 J groups, receiving a normocaloric diet and fluoride-free water for the 21-day duration. At the end of the experimental period (21 days), animals were euthanized by CO_2_ chamber and kidneys were harvested for analysis. For the histological assay, samples were fixed in 10% formalin buffered for 24 h, washed in running water for 12 h, and submitted to histological processing for birefringence and immunofluorescence analysis.

**Table 2:** In vivo group distribution according to each experimental condition

Group	Mice	Exposure scenario
Ctrl	C57BL/6 mice, normocaloric diet and deionized water	–
HG	C57BL/6J mice, high-fat diet, STZ, diabetic (> 200 mg/dL) and deionized water	Chronic hyperglycemia (glucose > 200 mg/dL) + 0mgF/L
F1 + HG	C57BL/6J mice, high-fat diet, STZ, diabetic (> 200 mg/dL) and 10 mgF/L NaF supplemented water	Chronic hyperglycemia (glucose > 200 mg/dL) + 1 mgF/L (aligned with WHO fluoridation guidelines)
F5 + HG	C57BL/6J mice, high-fat diet, STZ, diabetic (> 200 mg/dL) and 50 mgF/L NaF supplemented water	Chronic hyperglycemia (glucose > 200 mg/dL) + 5 mgF/L (representing endemic F areas with naturally elevated drinking water levels)

### F quantification in kidney tissue

F levels in kidney samples were quantified based on the protocol described by Pereira et al. (2018), with minor adaptations. Briefly, kidney samples (*n* = 6 per group) were analyzed in duplicate after overnight diffusion facilitated by hemathyldisiloxane (HMDS). The released F ions were measured using an ion-selective electrode (Accumet, #13-620-79) connected to a potentiometer (Orion Research, Model EA 940). F standards ranging from 100, 50, 10, and 1ppm F were prepared with the Orion™ ISE Calibration Standards (Orion 940907, Thermo Scientific) in triplicate standards and subjected to the same diffusion process as the tissue samples. Additional non-diffused standards, with identical F concentrations, were also prepared to verify recovery efficiency. The comparison of millivolt readings confirmed that F from diffused standards was fully recovered (recovery rate > 95%). Finally, the electrode potentials (mV) were converted into F concentrations (µg F) using a standard calibration curve with correlation coefficient of *r* > 0.99 (Pereira et al. 2013).

### Cell viability assay

Cell viability was assessed using the MTS assay (G5421, Promega Corporation), following the manufacturer recommendation. In brief, confluent cultures of M-1 cells were dissociated with 0.25% trypsin (25300054, Gibco) and seeded onto 96-well plates at a density of 5 × 10^3^ cells per well. Cells were treated under various conditions (Table 1), and cell viability was assessed at 24, 48 and 72 h of treatment. All assays were conducted with a minimum of 4 technical replicates, two different cell passages and 2 independent experiments. After 4 h of incubation with MTS, viability was determined by measuring absorbance at 490 nm by using a Microplate Spectrophotometer (Agilent Biotek Epoch) and data were analyzed using Gen6 software. Absorbance values were then normalized into % based on values of the Ctrl wells.

### Immunofluorescence assay

Immunofluorescence was performed to characterize cell morphology in M-1 cells using F-actin fluorophore, and KIM-1 marker in both murine kidney tissue and M-1 cells. For the in vitro assay, M-1 cells were cultured in 24-well plates in sterile coverslips at a density of 20 × 10^3^ cells/well following experimental treatments in Table 1. Then, cells were washed with 1xPBS and fixed with 4% formaldehyde for 15 min. Murine kidney paraffin slices of 4 micrometers were treated with steps of deparaffinization and rehydration, followed by antigen retrieval as previously described (Biguetti et al. 2021). Both cells and tissue samples were incubated for 2 h with anti-mouse KIM-1 primary antibody (AF1817, R&D System) at a 1:150 dilution. Following incubation, samples were treated with biotinylated FITC (1:150) secondary antibody (T2769, Thermo Fisher Scientific) for 2 h in the dark at room temperature. Negative controls from either cells or tissue samples without primary antibody were included to confirm the absence of non-specific binding. A blocking solution was applied prior to primary antibody incubation to minimize non-specific binding, and the primary antibody was diluted in PBS containing 1% BSA. After washing, DAPI was added to stain the nuclei (1:1000). For F-actin assessment and analysis of morphology, M-1 cells were stained with phalloidin (ab176753, Abcam) according to manufacturer’s instruction. After washing, DAPI was added to stain the nuclei (1:1000). Fluorescence microscopy analysis was performed using a Nikon NiU Eclipse Upright fluorescence microscope equipped with DAPI and FITC filters. Image acquisition parameters were standardized for all samples as follows: for DAPI, a 30 ms exposure time was used, with D-LEDI pad intensity set to 15%, and LUTs adjusted from 203 to 3694, for FITC, a 200 ms exposure time was used, with D-LEDI pad intensity set to 45%, and LUTs adjusted from 242 to 25,000. All images were captured and processed using these fixed parameters to ensure comparability across experimental groups and to minimize variations related to image acquisition. LUT and exposure setting were kept constant across analyzed fields and samples. Images were analyzed using NIS-Element software.

### Birefringence assay

Semi-serial kidney histological sections of 4 µm of thickness were obtained for birefringence qualitative analysis using Picrosirius red staining. All specimens were analyzed at 10 × and 20 × magnification through polarizing lenses coupled to a binocular inverted microscope (Leica DM IRB/E, Leica Microsystems Wetzlar GmbH, Wetzlar, Germany) and images were captured with a Leica Imaging Software (LAX, Leica, Leica Microsystems Wetzlar GmbH, Wetzlar, Germany). Analysis and procedures were done as previously (Biguetti, Cavalla, et al., 2018; Biguetti, Vieira, et al. 2018a, b), green birefringence color indicates thin fibers, yellow and red colors at birefringence indicate thick collagen fibers.

### Statistical analysis

All quantitative data was initially analyzed for distribution of normality by Shapiro-Wilk test, and homogeneity was assessed using Bartlett’s test for data, to select appropriated statistical parametric or non-parametric tests. Outliers were identified and removed prior to statistical testing using ROUT method with a false discovery rate of 5%. Data with normal distribution was analyzed using parametric test of One-Way ANOVA, while those with non-normal distribution were analyzed by non-parametric test of Kruskal-Wallis. The values of *p* < 0.05 were considered statically significant for all analysis. Test were conducted using GraphPad InStat Software version 10.0 for Windows (GraphPad, San Diego, CA).

## Results

### Glucose assessment

To confirm the establishment of DM type 2, fasting blood glucose was measured one week before and one week after STZ injection. Prior to induction, no significant differences were observed among groups (mean ± SD): Ctrl (139 ± 20.93 mg/dL), HG (140 ± 18.09 mg/dL), F1 + HG (136.2 ± 22.21 mg/dL), and F5 + HG (127.2 ± 12.33 mg/dL). As expected, after STZ administration, all diabetic groups HG (239.8 ± 22.36 mg/dL), F1 + HG (290 ± 70.94 mg/dL), and F5 + HG (289.1 ± 73.74 mg/dL) exhibited significantly higher glucose levels compared with the Ctrl group (126.7 ± 18.19 mg/dL, *p* < 0.05; Supplemental Fig. 1). Analyses were performed with group size ranging from *n* = 8–10.

### Body weight

At week 0, no significant differences were observed among groups (mean ± SD): Ctrl (21.20 ± 1.03 g), HG (21.77 ± 1.42 g), F1 + HG (19.81 ± 1.68 g), and F5 + HG (19.81 ± 3.082 g) (Supplementary Fig. 3A). After 8 weeks on the high-fat diet, all groups exposed to this diet, HG (30.57 ± 1.279 g), F1 + HG (30.84 ± 1.027 g), and F5 + HG (30.27 ± 0.907 g), showed significantly higher body weight compared with the Ctrl group (26.51 ± 1.279 g, *p* < 0.05; Supplementary Fig. 3B) that consumed a normocaloric diet. By week 12, following the treatment period, no significant differences in body weight were observed among any of the groups: Ctrl (29.07 ± 1.864 g), HG (27.02 ± 2.794 g), F1 + HG (29.01 ± 1.346 g), and F5 + HG (29.13 ± 2.029 g) (Supplementary Fig. 3C).

### F quantification in kidney tissue

Mean (± SD) kidney F levels for Ctrl, HG, F1 + HG, and F5 + HG were 0.052 ± 0.0204 µg/g, 0.40 ± 0.115 µg/g, 0.93 ± 0.0320 µg/g, and 0.40 ± 0.174 µg/g respectively. F levels observed in the F5 + HG group were significantly higher when compared with the Ctrl, HG, and F1 + HG groups (*p* < 0.05; Supplementary Fig. 4).

### Cell viability assay

First, we evaluated whether the increased osmolarity resulting from the addition of glucose to the media could independently affect M1-cell viability. To investigate this, we utilized mannitol as an osmolarity control, as it does not affect glycolytic pathways (Mei et al. 2022). We tested two different concentrations, addition of 5mM and 7.5 mM, and evaluated the results for 24 h. No significant differences were observed between the Ctrl and the MNT 5mM or MTN 7.5 mM groups, however, the groups MNT 5 mM and MNT 7.5 mM were significantly different from each other (Supplemental Fig. 2A). At 48 h no significant difference between the Ctrl group and the MNT 7.5 mM (Supplemental Fig. 2B). Lastly, for the period of 72 h it wasn’t observed a significant difference between the experimental groups (Supplemental Fig. 2C). Based on these results, it was determined to continue the experiments with the concentration of adding up 5mM added glucose to the media for the HG groups.

**Fig. 1:**
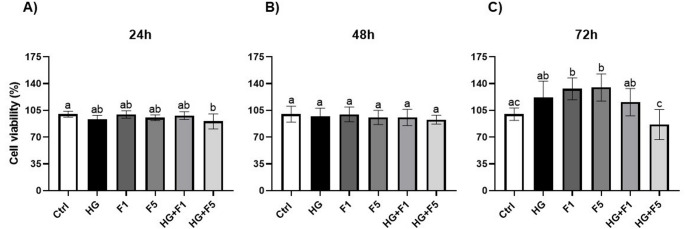
Viability assay of M-1 cells. **A** MTS analysis of M-1 cells from the experimental groups Ctrl, HG, F1, F5, F1 + HG and F5 + HG over a 24-hour period, **B** MTS analysis of M-1 cells from the experimental groups Ctrl, HG, F1, F5, F1 + HG and F5 + HG over a 48-hour period, **C** MTS analysis of M-1 cells from the experimental groups Ctrl, HG, F1, F5, F1 + HG and F5 + HG over a 72-hour period. Data are presented as mean (%) ± SD of three independent experiments performed in quadruplicate (*n* = 4). Statistical analysis was performed using ANOVA *p* < 0.05 was considered significant. For visual representation, the groups were assigned letter notations based on their statistical differences, groups sharing at least one common letter are not significantly different from each other

Next, we evaluated the influence of HG and/or F on M-1 cell viability at 24 h, 48 h, and 72 h. The MTS assay at 24 h revealed a significant reduction in cell viability in the F5 + HG groups (90.53 ± 9.84%) compared with the Ctrl (99.99 ± 3.77%) and HG (93.16 ± 5.25%) groups (*p* < 0.05; Fig. 1A). At 48 h, no statistical differences were observed among groups (Fig. 1B). At 72 h, cell viability in the Ctrl group (100 ± 8.17%) was significantly lower compared with the F1 (133.2 ± 14.21%) and F5 (134.9 ± 17.78%) groups (*p* < 0.05; Fig. 1C). The combination of F5 + HG (86.42 ± 19.63%) significantly decreased M1-cell viability compared with all treated groups, except for Ctrl (*p* < 0.05). The viability of the F1 group (133.2 ± 14.21%) was higher than both Ctrl (100 ± 8.17%) and F5 + HG (86.42 ± 19.63%) groups (*p* < 0.05), while the F5 group (134.9 ± 17.78%) also showed increased viability compared with Ctrl and F5 + HG (*p* < 0.05). In addition, the F1 + HG group (115.0 ± 17.87%) displayed a significant increase compared with F5 + HG (86.42 ± 19.63%) and HG (121.8 ± 21.58%) groups (*p* < 0.05). Based on these findings, the 72 h time point was selected for subsequent immunofluorescence assays, as it provided the most pronounced differences in cell viability across groups and was more relevant to evaluate potential chronic effects of exposure to HG and F.

### Morphological assessment by F-actin staining with phalloidin

**Fig. 2:**
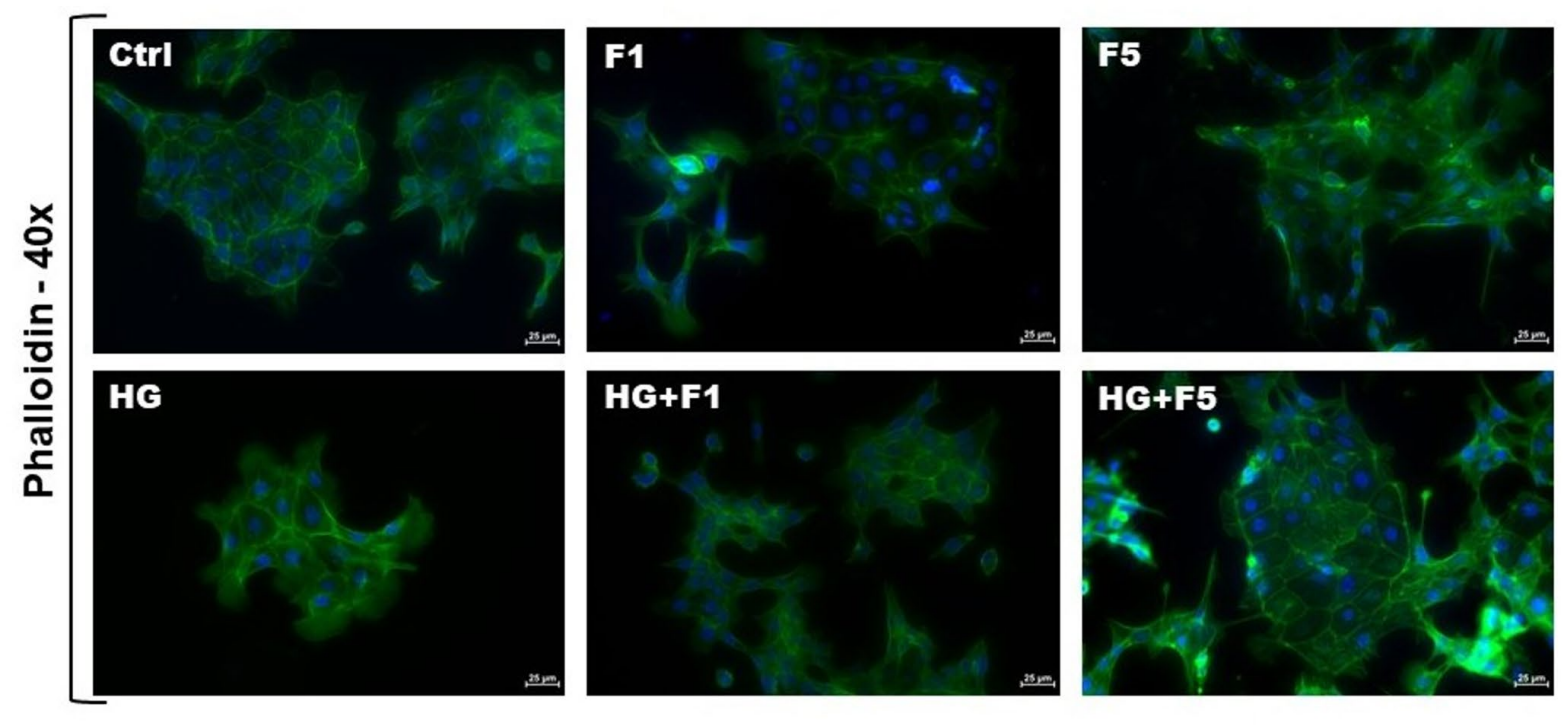
Phalloidin staining (green) of actin filaments and DAPI staining (blue) in M-1 kidney cells from the experimental groups Ctrl, HG, F1, F5, F1 + HG and F5 + HG over a 72-hour period. Images were captured using a camera coupled to an upright fluorescence microscopy with a 40x objective. All exposure settings were the same across the samples. The scale bar represents 25 μm (40x)

The morphology of M1-cell colonies was observed by staining actin filaments in M-1 renal duct epithelial cells, revealing filamentous cytoskeletal structures across all treatment groups. It was possible to notice the formation of clusters as a main feature of this cell type, displayed in all groups. In the Ctrl group, actin filaments appear well-organized, forming a dense, round network throughout the cytoplasm. The F1 group displayed cells with a morphology like the Ctrl group; however, some cells exhibited a more elongated shape, consistent with longitudinal stretching and these cells did not form as large clusters as those with a rounder morphology. In the F5 and HG groups, large clusters were observed but still exhibiting longitudinal stretching. In the F1 + HG and F5 + HG groups, clusters were formed, and the cells displayed an elongated shape (Fig. 2).

### Immunofluorescence of KIM-1 in vitro and in vivo

**Fig. 3:**
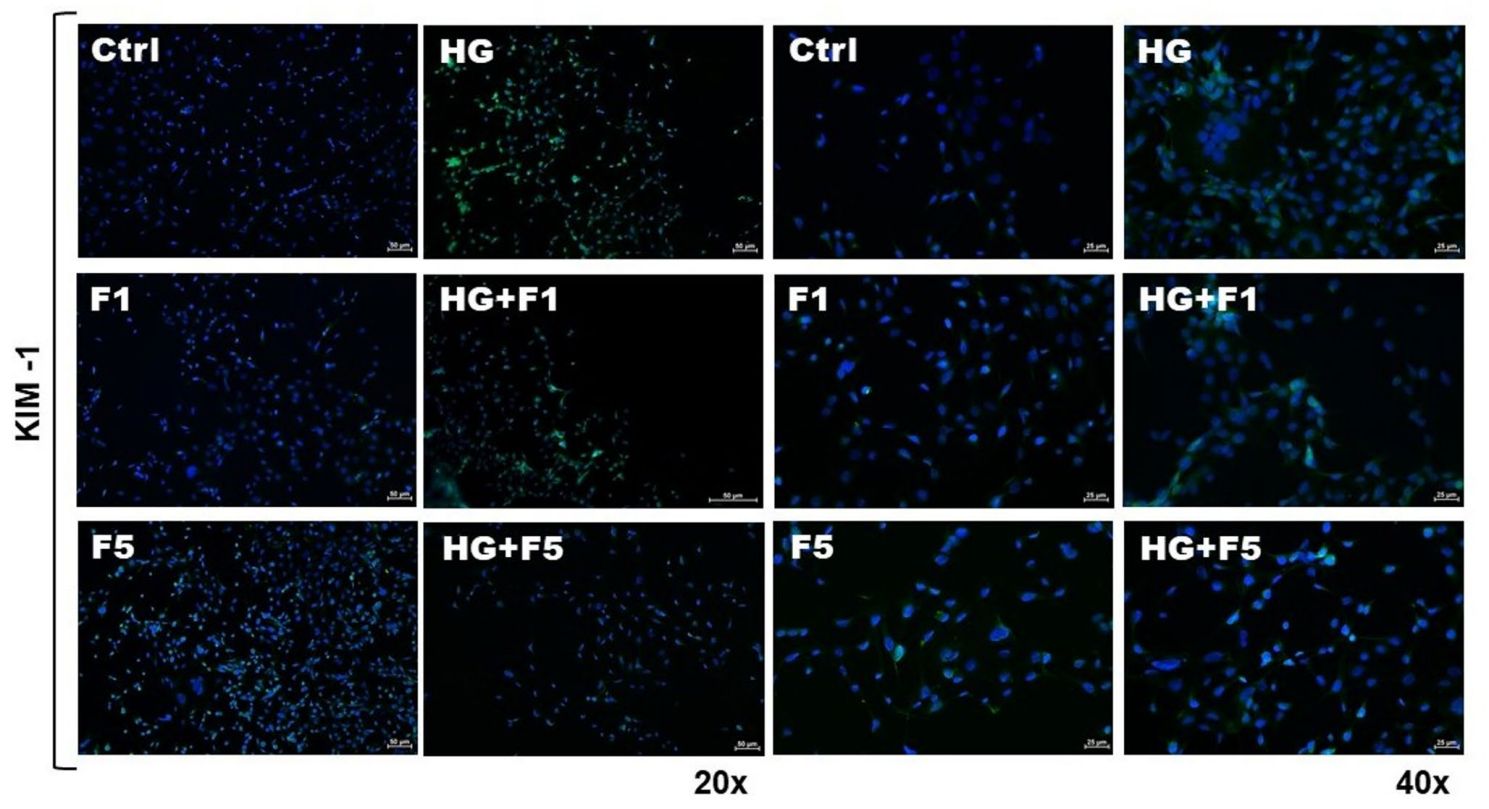
KIM-1 staining (green) and DAPI staining (blue) in M-1 kidney cells from the experimental groups Ctrl, HG, F1, F5, F1 + HG and F5 + HG over a 72-hour period. Images were captured using a camera coupled to an upright fluorescence microscopy with a 20x and 40x objective. LUT and exposure setting were kept constant across analyzed fields and samples. The scale bar represents 50 μm (20x) and 25 μm (40x)

The immunofluorescence for KIM-1 in M-1 cells revealed differential signal expression across the experimental groups. In the Ctrl and F1 groups, KIM-1 expression was very low, with faint fluorescence signal. The F5 group exhibited a slightly higher intensity in KIM-1 fluorescence. In contrast, both HG and F1 + HG groups displayed marked fluorescence intensity compared to the Ctrl group. Interestingly, the F5 + HG group showed KIM-1 expression comparable to that of the Ctrl group (Fig. 3).

**Fig. 4:**
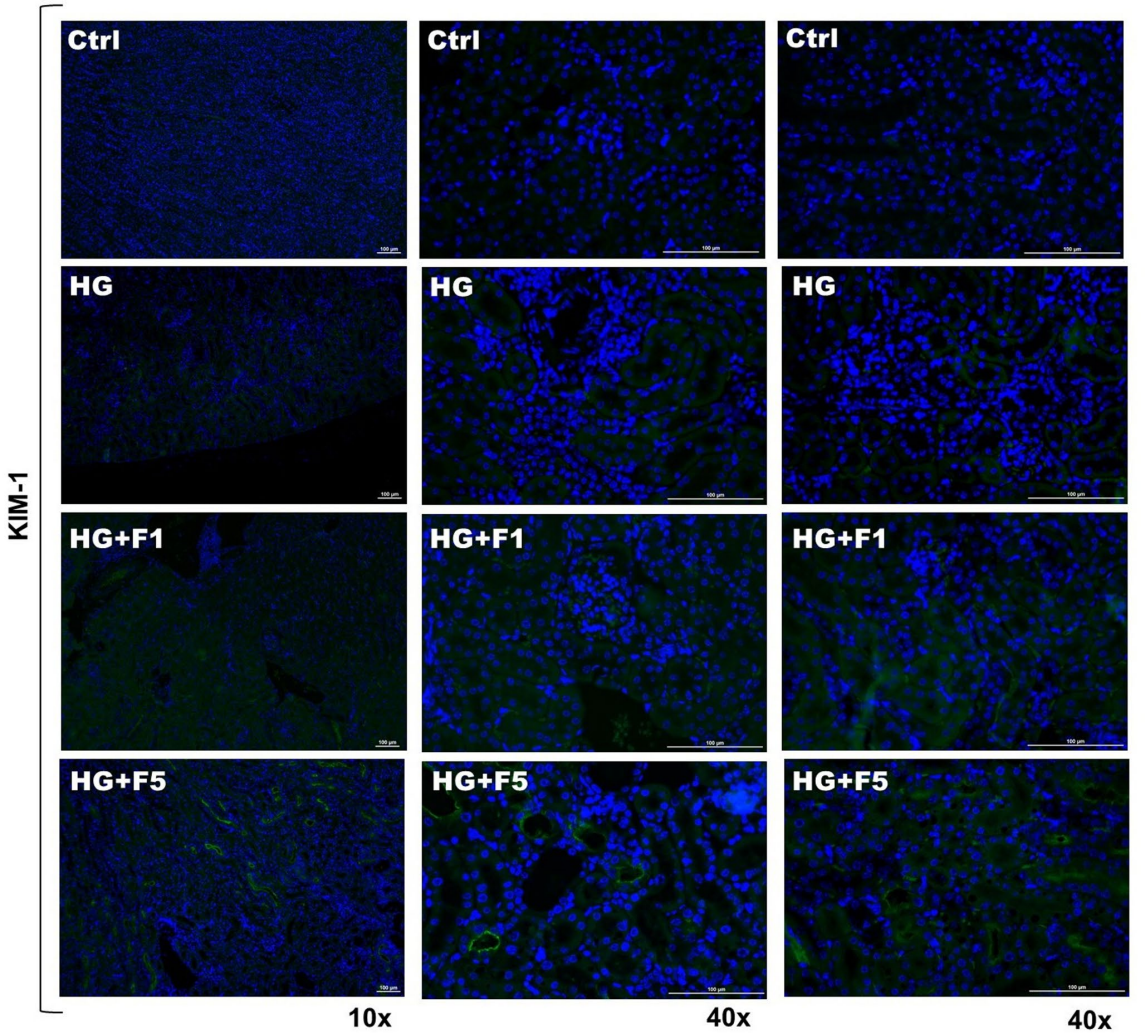
KIM-1 staining (green) and DAPI staining (blue) in kidney samples of diabetic or non-diabetic C57BL/6 and C57BL/6J mice of the following experimental groups: Ctrl, HG, F1 + HG and F5 + HG. Representative images were captured using an immunofluorescence microscopy with 10x and 40x objectives. Images at 40x are two representative pictures of different areas of the cortex of the kidney. LUT and exposure setting were kept constant across analyzed fields and samples. The scale bar represents 100 μm (10x) and 100 μm (40x)

Immunofluorescence staining of KIM-1 in kidney tissue samples revealed distinct patterns of signal intensity and distribution across the experimental groups. In the Ctrl group, KIM-1 expression was low intensity, with only very faint fluorescent signal. Both the HG and F1 + HG groups exhibited similar fluorescence patterns, with a faint KIM-1 signal present in the proximal tubules. However, the F5 + HG group was the only one showing a distinct and strongly positive KIM-1 staining, with a noticeable fluorescence around the edges of the proximal tubules (Fig. 4).

### Birefringence analysis for collagen deposition

**Fig. 5:**
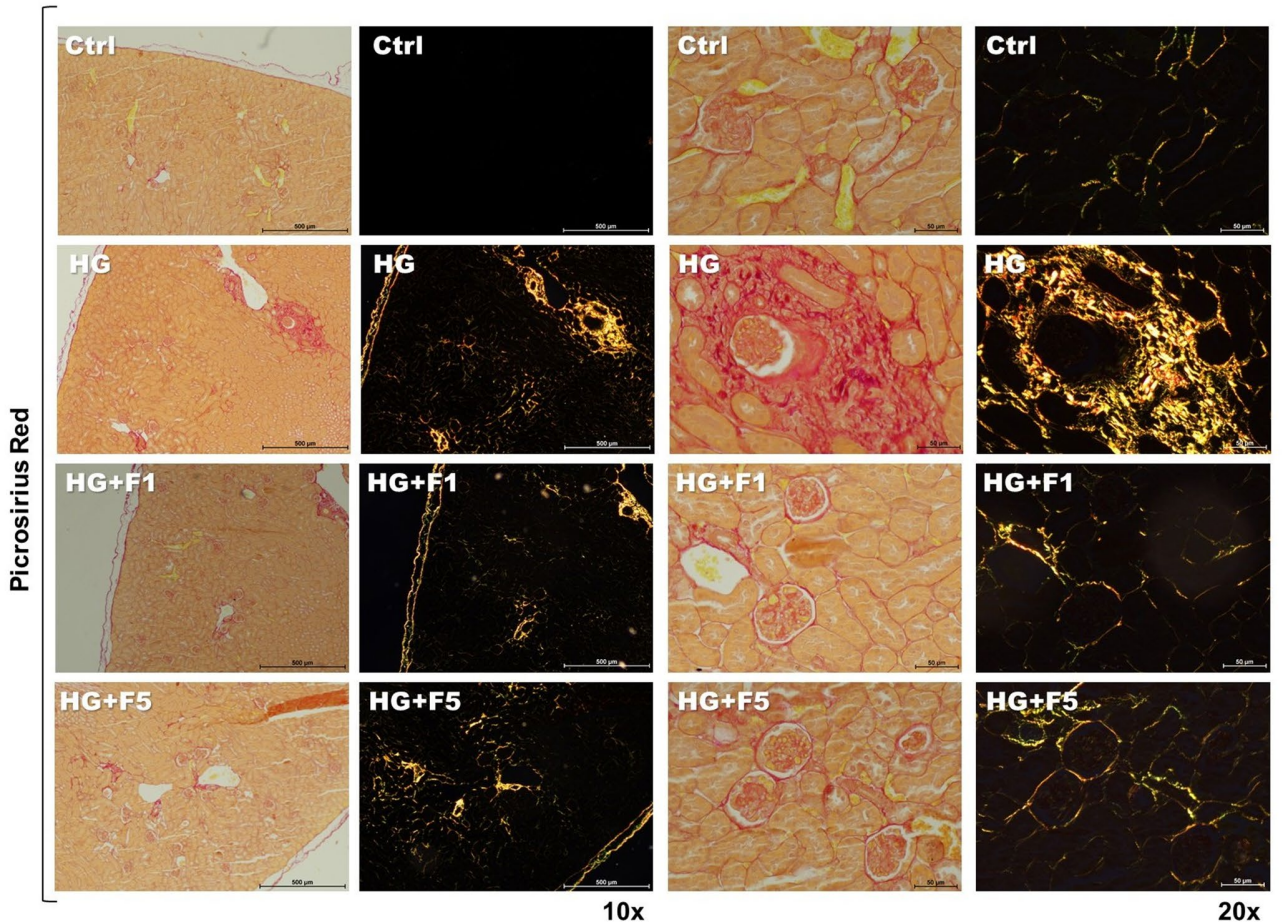
Picrosirius red staining of collagen I and III was examined by polarized light microscopy in kidney samples of diabetic and non-diabetic C57BL/6 and C57BL/6j mice of the following experimental groups: Ctrl, HG, F1 + HG and F5 + HG. Collagen type I fibers appear yellowish orange, to red, whereas collagen type III fibers appear as green to yellowish green against a black background. Representative images were captured using a binocular inverted microscope with 10x and 20x objectives. All exposure setting were the same across the samples. The scale bar represents 500 μm (10x) and 50 μm (20x)

Birefringence analysis by Picrosirius red staining revealed distinct patterns of collagen expression and renal tissue morphology across the experimental groups. The Ctrl group showed normal renal morphology, characterized by well-preserved glomerular and tubular structures, with minimal collagen deposition in the interstitial and periglomerular regions. These groups served as the baseline for normal kidney architecture. In contrast, the HG group displayed significant morphological alteration, particularly an elevated concentration of collagen around the glomeruli, indicative of fibrosis. Interestingly, the F1 + HG and F5 + HG groups showed renal morphology similar to that of the Ctrl group, with no significant increase in collagen deposition around the glomeruli (Fig. 5).

## Discussion

Water fluoridation stands as one of public health’s greatest achievements, by maintaining optimal F levels, communities worldwide can maximize its oral health benefits while safeguarding overall wellbeing, striking a crucial balance between prevention and safety (Buzalaf 2018). Our experimental design included varying F concentration for both cell culture and animal model, selected to reflect levels relevant to human studies on fluoridated water and fluorosis when those exceed recommended limits by WHO and ADA (Ribeiro et al. 2017). Specifically, concentrations were based on regions with low to normal F levels (1–2.4 mgF/L) and areas with elevated F levels (10–80 mgF/L)(Mendoza-Schulz et al. 2009). While numerous studies have focused on hard tissues (Arakawa et al. 2009; Otsuki et al. 2005), there is a growing need to understand the effects of chronic F exposure on soft tissues, particularly the kidneys which play a crucial role in F absorption and excretion (Arakawa et al. 2009; Otsuki et al. 2005). This is especially relevant for populations susceptible to developing CKD, such as adults with long term history of type 2 DM, who may experience altered F metabolism and increased vulnerability to its potential toxicity (Jiménez-Córdova et al. 2018). Indeed, various factors, including acid–base balance, physical activity, nutritional status, age, and genetic makeup, may influence F metabolism and modulate its retention and potential toxicity (Buzalaf 2018). Therefore, this study aimed to investigate the potential adverse effects of F on M-1 renal tubular epithelial cells exposed to a simulated hyperglycemic environment, as well as its impact on the kidney of type 2 diabetic C57BL/6 J mice. We assessed the impact of F on M-1 cell viability, morphology, and the expression of the injury marker KIM-1 in vitro and in vivo, we also evaluated in vivo collagen deposition using birefringence analysis by Picrosirius red staining. To our knowledge this is the first study to investigate the effect of simultaneous exposure of F and HG in kidney M-1 cells and diabetic mouse samples.

We first examined HG effects on M-1 renal tubular epithelial cell viability and morphology. These cells primarily use SGLT2 transporters for glucose reabsorption (Ghezzi et al. 2018), during hyperglycemia SGLT2 upregulation increases glucose uptake, potentially leading to glucotoxicity, oxidative stress, and tubular damage (Gou et al. 2016). Previous studies show divergent responses to HG, mesangial cells exhibit increased viability/proliferation (Chen et al. 2016; Peres et al. 2017), while epithelial cells maintain stable viability. Our MTS assay (24–72 h) confirmed this pattern in M-1 cells, showing no significant viability changes when compared to the Ctrl group. This cell-type specificity likely stems from differential glucose transporter expression, epithelial cells express SGLT2, whereas mesangial cells express SLC2A1(Peres et al. 2017). Building on these findings, we next investigated the effects of HG and/or F exposure on M-1 tubular epithelial cells, focusing on viability and morphology. In this study, both F-treated groups (F1 and F5) showed increased viability when compared to the Ctrl group, with no significant difference between them.

It is also worth to note that F is known to impair mitochondrial functions and ATP production, leading to shifts in cellular metabolism (Araujo et al. 2019). F-induced cytotoxicity has been extensively linked to oxidative stress and inflammation (Kobayashi et al. 2009). Excess F promotes the generation of reactive oxygen species (ROS), mitochondrial membrane depolarization, and lipid peroxidation, which compromise cell integrity (Barbier et al., 2010; Korkmaz et al. 2022). In addition, F can activate pro-inflammatory signaling pathways such as NF-kB and MAPK, enhancing the production of cytokines like TNF-α and IL-6 (Araujo et al. 2019; Shanthakumari et al. 2004). In parallel, F exposure may alter cellular bioenergetics by reducing glycolytic flux and stimulating glutamine metabolism to sustain ATP synthesis under stress conditions (Lei et al. 2019; Otsuki et al. 2005). These adaptative metabolic responses could explain the higher viability observed in the low-F groups, reflecting a glutamine-dependent survival mechanisms. These results are aligned with previous studies showing that low F concentrations can promote proliferation, while higher levels typically reduce viability depending on cell type and context (Arakawa et al. 2009; Gutowska et al. 2016; Korkmaz et al. 2022; Lei et al. 2019; Otsuki et al. 2005; Santesso et al. 2021; Wei et al. 2014). Under HG conditions, the F1 + HG group showed significantly higher viability than the F5 + HG group, suggesting that HG may modulate the effect of F at a higher concentration, this finding is in agreement with previous studies that F can interfere in the glycolytic pathway (Buzalaf 2018). However, the F5 + HG group did not differ statistically from the Ctrl group, indicating that the combination of high F concentration and HG does not impair cell viability. Additionally, the F1 + HG group showed no statistical difference in viability to the F1 and F5 groups, with all showing higher values compared to the Ctrl group, in agreement with previous studies (Arakawa et al. 2009; Gutowska et al. 2016; Korkmaz et al. 2022; Lei et al. 2019; Otsuki et al. 2005; Santesso et al. 2021; Wei et al. 2014).

As most effects were observed at 72 h, KIM-1 and morphology experiments were conducted at this time point. KIM-1 is a membrane protein minimally expressed in healthy kidneys, but upregulated in injured proximal tubules, thus serves as a marker of tubular damage (Humphreys et al. 2013; Ichimura et al. 2004). In addition, it is noted that in acute injuries KIM-1 may be beneficial helping with repair, suggesting an attempt by the cells to adapt in response to damage, however in chronic stages it may contribute to fibrosis (Huo et al. 2010). Gou, et al. (2016) showed that HG increases KIM-1 expression, indicating hyperglycemia-induced damage. Similarly, our cell model showed elevated KIM-1 in the HG group compared to the Ctrl. However, this was not reflected in the animal model, where the HG group showed little to no KIM-1 expression. In the present study, HG conditions increased KIM-1 expression in vitro, as observed in the HG and F1 + HG groups. Interestingly, the F5 + HG group exhibited reduced KIM-1 expression levels. Considering the cell viability results, it is possible to hypothesize that the HG and F1 + HG groups showed a higher KIM-1 expression because both HG and F appeared to have a greater impact in these conditions, whereas the F5 + HG group showed no difference from the Ctrl group. We also hypothesize that the increased KIM-1 expression in the HG and F1 + HG groups may be related to an acute cellular response, as previously mentioned, where KIM-1 upregulation could support repair mechanisms (Huo et al. 2010). Therefore, the higher expression observed in these groups is likely correlated with the findings of the cell viability. In addition, the F5 + HG group, some preservation of cell morphology was observed in the phalloidin staining assay.

As observed in the in vivo KIM-1 assay, the results diverged from those obtained in the cellular model, as only the F5 + HG group exhibited a strong positive expression of KIM-1. This divergence reflects the fundamental biological differences between in vitro and in vivo systems. In cell culture, F directly interacts with renal epithelial cells under tightly controlled conditions, triggering acute stress responses such as mitochondrial dysfunction, oxidative stress, and enzyme inhibition (Johnston and Strobel 2020). On the other hand, in the in vivo model, F exposure is modulated by complex systemic factors including absorption, distribution, metabolism, excretion, hormonal control, and immune regulation, which collectively influence renal susceptibility and repair capacity (Zohoori and Buzalaf 2022). In addition, the speciation and compartmentalization of F in vivo, through its binding to plasma proteins, ions, or bone tissue can reduce its free concentration and biological reactivity, an effect absent in isolated cell systems (Buzalaf 2018; Buzalaf et al. 2001; Kobayashi et al. 2009). These systemic buffering mechanisms, along with compensatory antioxidant and anti-inflammatory defenses such as catalase and glutathione peroxidase activation, as demonstrated in in vivo F exposure models (Morales-González et al. 2010) may attenuate early markers of tubular injury like KIM-1 in vivo. Collectively, this divergence underscores the dose- and time-dependent nature of F effects and indicates that KIM-1 upregulation may require prolonged exposure, hyperglycemic conditions, and a high F burden to become evident in vivo.

Finally, to further explore the structural changes in collagen matrix associated with HG and/or F treatment in vivo, we utilized birefringence analysis by Picrosirius staining to detect patterns of collagen deposition. Indeed, it is well known that in diabetic kidney disease there is an increased deposition of collagen content, following kidney injury, cells release inflammatory signal and extracellular matrix (ECM) proteins to promote healing, however, when damage exceeds repair, ECM accumulation disrupts tissue structure resulting in fibrosis that may lead to impair function of the organ (De Gregorio et al. 2025). In the present study, the Ctrl group showed normal renal morphology with minimal collagen deposition. In contrast, the HG group exhibited increased collagen around the glomeruli, indicating early fibrosis. Al Omirreni, et al. (2010) administered intraperitoneal NaF (5–50 mg/kg) to Wistar rats and observed reduced fibrosis in hard tissues. Similarly, Siddiqi (2012) reported decreased renal collagen in rats treated with 10 and 20 mg/kg NaF. Consistent with these findings, our study also showed reduced collagen deposition in F-treated groups, regardless of dose. The F1 + HG and F5 + HG groups displayed renal morphology similar to the Ctrl group, suggesting an inhibitory effect of F against diabetes-induced fibrosis. While fluorides influence on collagen in hard tissues is well documented, these findings support its potential to reduce collagen deposition in soft tissues such as the kidneys (Al Omireeni et al. 2010).

The contrasting findings between the reduction of collagen and the increase in KIM-1 expression in the animal model (F5 + HG group) is an interesting observation. This could suggest that F may affect different parts of the kidney in distinct ways. While collagen deposition is primarily associated with glomerular alterations and extracellular matrix remodeling (Al Omireeni et al. 2010), KIM-1 expression mainly reflects on proximal tubular injury (Abid Khan et al. 2019). Therefore, F could exert a protective effect against fibrosis while simultaneously causing tubular stress under hyperglycemic conditions. Among the limitations of this study, we highlight the absence of direct functional analyses of cellular metabolism, which could provide deeper insights into the bioenergetics mechanisms involved. In addition, the immunofluorescence and birefringence analyses were qualitative in nature, and no quantification of fluorescence intensity or collagen area was performed, which could strength the statistical robustness of the findings. In addition, future studies should consider assessing additional markers of tubular injury and inflammation to gain a more comprehensive understanding of the interplay between F exposure and hyperglycemia.

From a public health perspective, our findings emphasize the importance of maintaining F exposure within optimal limits, particularly among individuals with metabolic disorders such as T2DM. The observed modulation of renal marker under hyperglycemic conditions suggests that diabetic populations may exhibit altered F handling, increasing susceptibility to nephrotoxic effects when environmental or dietary F levels exceed recommended thresholds. Therefore, these data reinforce the need for ongoing monitoring of F concentrations in drinking water and for considering metabolic status in risk assessment and fluoridation policies. Ensuring appropriate F intake remains essential to balance its well-established dental benefits with systemic safety.

## Conclusions

**Fig. 6:**
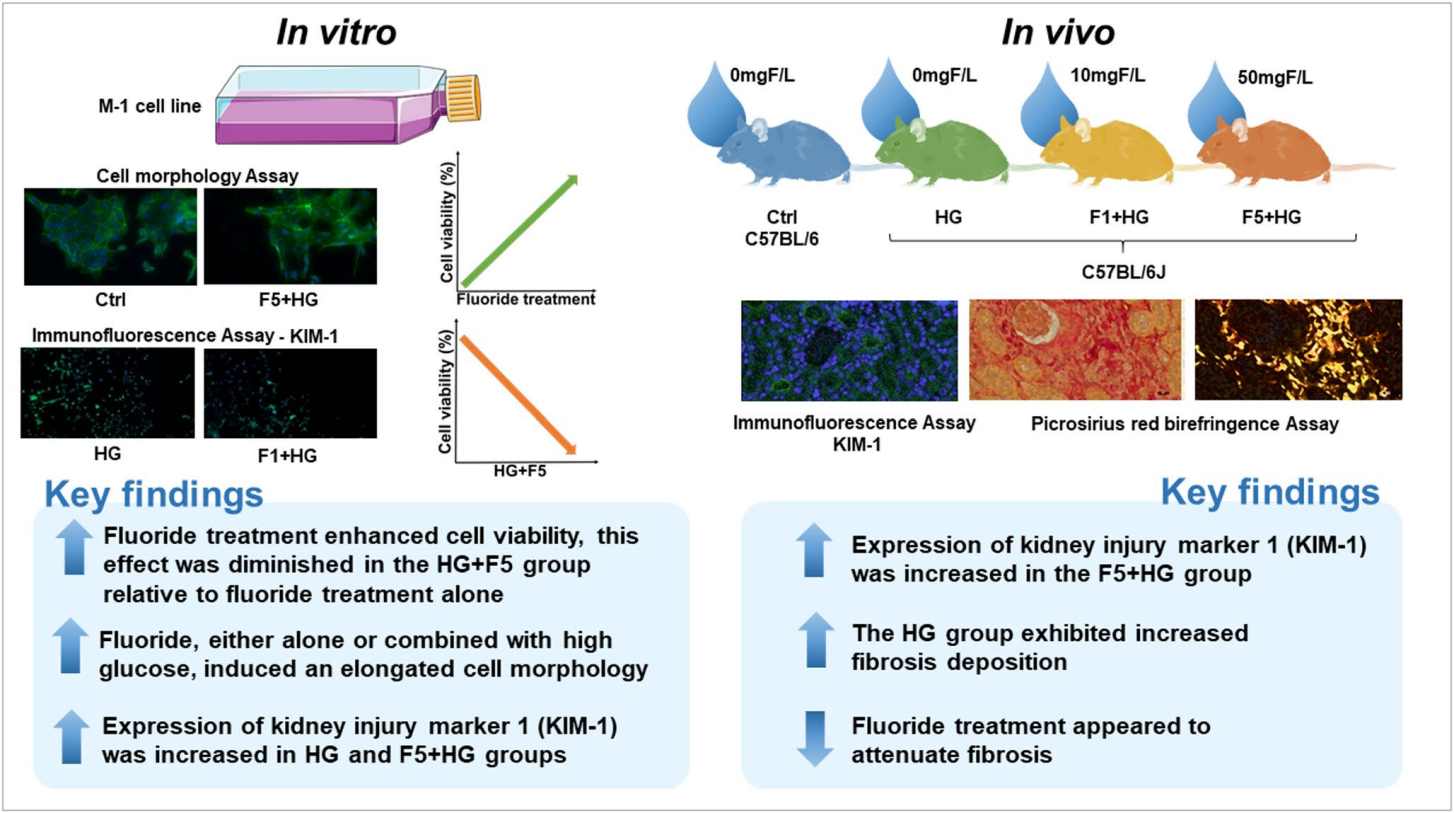
Visual summary of key experimental findings. In the in vivo assay, immunofluorescence analyses demonstrated positive KIM-1 staining exclusively in the F5 + HG group. Fibrosis was distinctly detected in the HG group by picrosirius red birefringence, while groups treated with F exhibited minimal fibrotic changes. These findings suggest that F treatment may exert an inhibitory effect on fibrosis development. For the in vitro experiments, treatment with fluoride alone (F1 or F5) significantly increased cell viability relative to the Ctrl group, whereas combined F5 + HG exposure led to a reduction in viability compared to the F1 and F5. Immunofluorescence data showed positive KIM-1 staining only in the HG and F1 + HG groups. The phalloidin staining revealed characteristic morphological alterations among all experimental groups (HG, F1, F5, F1 + HG, and F5 + HG), with cells displaying a more elongated morphology compared to the Ctrl group

Given all benefits of F in oral health and its prevalence in water system worldwide, it is important to investigate its potential interactions with other organs systems. This study explored the effects of F on renal tubular epithelial cells under hyperglycemic conditions, both in vitro and in vivo. The results showed that in vitro F alone and combined with HG induced changes in cell viability, morphology, and KIM-1 expression, suggesting an adaptive metabolic response to fluoride-induced stress. Diabetic mice exhibited early renal fibrosis, which was mitigated by F treatment, indicating a possible inhibitory role against diabetes-induced renal damage (See Fig. 6 for a graphical summary of the results). However, elevated expression of KIM-1 in F-treated group highlighted a potential tubular injury in combination with DM status at higher dose. These findings underscore the dual role of F in renal health, emphasizing the need for careful consideration of exposure levels, particularly in populations at risk of CKD in regions endemic fluorosis.

## Supplementary Information

Below is the link to the electronic supplementary material.

Supplementary Material 1. Supplemental Fig. 1. Fasting blood glucose levels before and after DM type 2 induction with STZ. Fasting blood glucose was measured one week prior to STZ and one week after STZ injection in Ctrl, HG, F1 + HG, and F5 + HG groups. Data are presented as mean ± SD, with group sizes ranging from *n* = 8–10 animals. Statistical analysis was performed using ANOVA *p* < 0.05 was considered significant. For visual representation, the groups were assigned letter notations based on their statistical differences, groups sharing at least one common letter are not significantly different from each other.

Supplementary Material 2. Supplemental Fig. 2. Viability assay. (A) MTS analysis M-1 cells treated with mannitol at the concentration of 5mM and 7.5mM over a period of 24 h. (B) MTS analysis M-1 cells treated with mannitol at the concentration of 5mM and 7.5mM over a period of 48 h. (C) MTS analysis M-1 cells treated with mannitol at the concentration of 5mM and 7.5mM over a period of 72 h. Values are expressed as mean (%) ± SD of two independent experiments. For visual representation, the groups were assigned letter notations based on their statistical differences (*p* < 0.05), groups sharing at least one common letter are not significantly different from each other.

Supplementary Material 3. Supplemental Fig. 3. Body eight of the Ctrl, HG, F1 + HG, and F5 + HG groups throughout the experimental period. (A) Week 0, (B) Week 8, when STZ was administered intraperitoneally to induce diabetes, and (C) Week 12, corresponding to the end of the experiment. Data are presented as mean ± SD, with group sizes ranging from *n* = 8–10 animals. Statistical analysis was performed using ANOVA *p* < 0.05 was considered significant. For visual representation, the groups were assigned letter notations based on their statistical differences, groups sharing at least one common letter are not significantly different from each other.

Supplementary Material 4. Supplemental Fig. 4. Fluoride (F) quantification in kidney tissue from the Ctrl, HG, F1 + HG, and F5 + HG groups. Data are presented as mean ± SD, with group sizes ranging from *n* = 6 animals. Statistical analysis was performed using ANOVA *p* < 0.05 was considered significant. For visual representation, the groups were assigned letter notations based on their statistical differences, groups sharing at least one common letter are not significantly different from each other.

## Data Availability

No datasets were generated or analysed during the current study.
