# Supplementary figures and images for: Combined effects of chronic high glucose and fluoride exposure on kidney cells: exploratory in vitro and in vivo study
Source: J Mol Histol. 2026 Jan 12;57(1):42. doi: 10.1007/s10735-025-10670-6 (PMC12795923; doi:10.1007/s10735-025-10670-6)

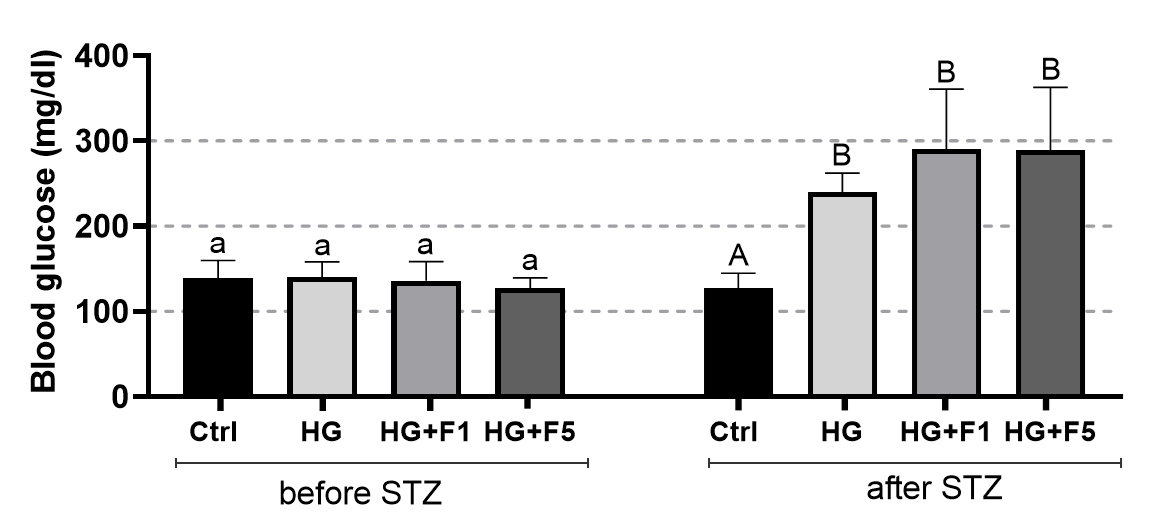

Supplement: Supplementary file 1 — Supplementary Material 1. Supplemental Fig. 1. Fasting blood glucose levels before and after DM type 2 induction with STZ. Fasting blood glucose was measured one week prior to STZ and one week after STZ injection in Ctrl, HG, F1 + HG, and F5 + HG groups. Data are presented as mean ± SD, with group sizes ranging from n = 8–10 animals. Statistical analysis was performed using ANOVA p < 0.05 was considered significant. For visual representation, the groups were assigned letter notations based on their statistical differences, groups sharing at least one common letter are not significantly different from each other. [file 10735_2025_10670_MOESM1_ESM.tif]

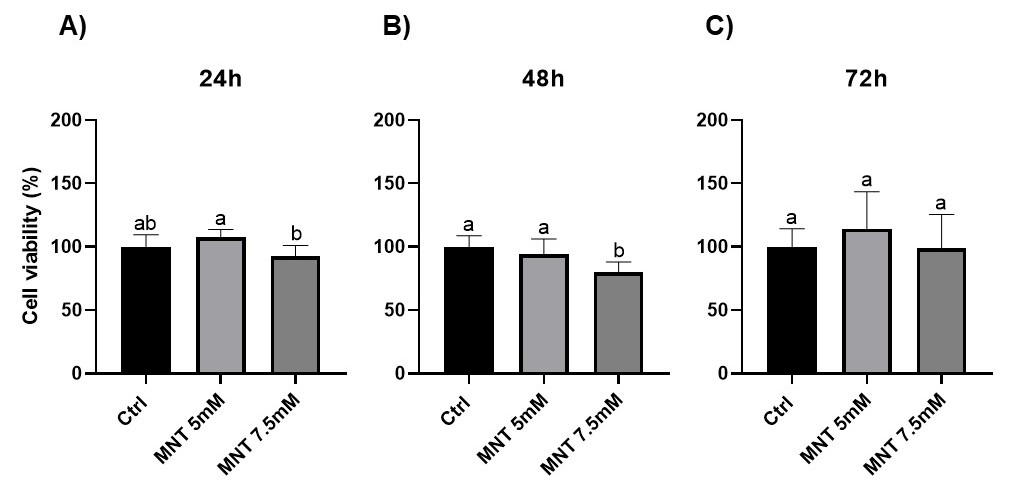

Supplement: Supplementary file 2 — Supplementary Material 2. Supplemental Fig. 2. Viability assay. (A) MTS analysis M-1 cells treated with mannitol at the concentration of 5mM and 7.5mM over a period of 24 h. (B) MTS analysis M-1 cells treated with mannitol at the concentration of 5mM and 7.5mM over a period of 48 h. (C) MTS analysis M-1 cells treated with mannitol at the concentration of 5mM and 7.5mM over a period of 72 h. Values are expressed as mean (%) ± SD of two independent experiments. For visual representation, the groups were assigned letter notations based on their statistical differences (p < 0.05), groups sharing at least one common letter are not significantly different from each other. [file 10735_2025_10670_MOESM2_ESM.tiff]

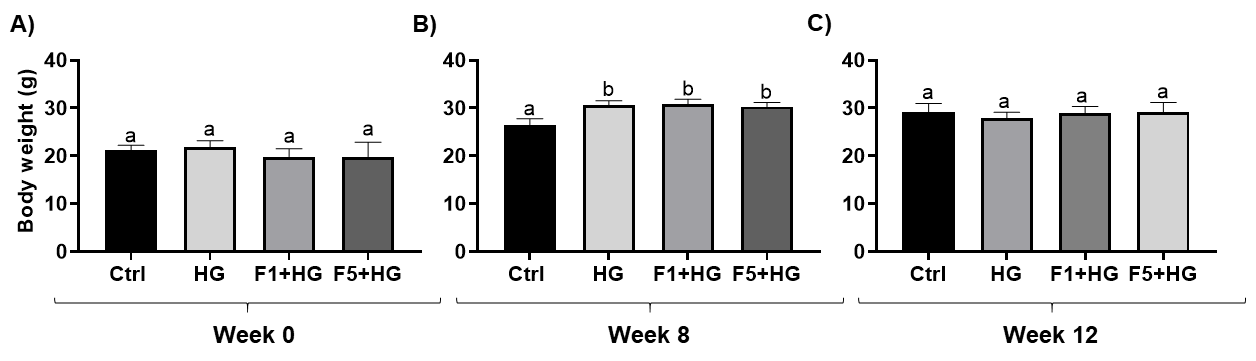

Supplement: Supplementary file 3 — Supplementary Material 3. Supplemental Fig. 3. Body eight of the Ctrl, HG, F1 + HG, and F5 + HG groups throughout the experimental period. (A) Week 0, (B) Week 8, when STZ was administered intraperitoneally to induce diabetes, and (C) Week 12, corresponding to the end of the experiment. Data are presented as mean ± SD, with group sizes ranging from n = 8–10 animals. Statistical analysis was performed using ANOVA p < 0.05 was considered significant. For visual representation, the groups were assigned letter notations based on their statistical differences, groups sharing at least one common letter are not significantly different from each other. [file 10735_2025_10670_MOESM3_ESM.tif]

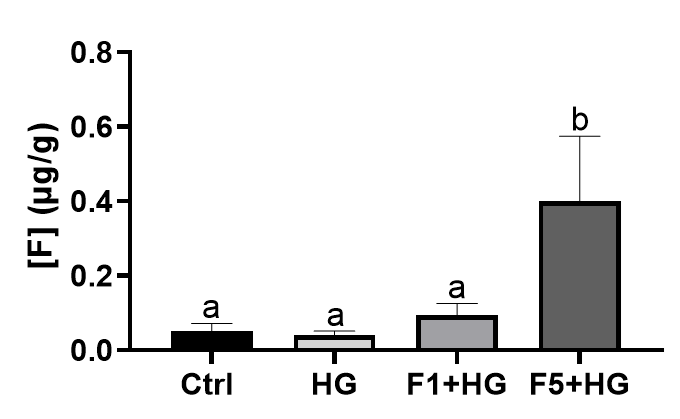

Supplement: Supplementary file 4 — Supplementary Material 4. Supplemental Fig. 4. Fluoride (F) quantification in kidney tissue from the Ctrl, HG, F1 + HG, and F5 + HG groups. Data are presented as mean ± SD, with group sizes ranging from n = 6 animals. Statistical analysis was performed using ANOVA p < 0.05 was considered significant. For visual representation, the groups were assigned letter notations based on their statistical differences, groups sharing at least one common letter are not significantly different from each other. [file 10735_2025_10670_MOESM4_ESM.tif]
